# Compressive Force Spectroscopy: From Living Cells to Single Proteins

**DOI:** 10.3390/ijms19040960

**Published:** 2018-03-23

**Authors:** Jiabin Wang, Meijun Liu, Yi Shen, Jielin Sun, Zhifeng Shao, Daniel Mark Czajkowsky

**Affiliations:** 1 Shanghai Center for Systems Biomedicine, Shanghai Jiao Tong University, Shanghai 200240, China; wjb0221@sjtu.edu.cn (J.W.); jlsun@sjtu.edu.cn (J.S.); 2 School of Biomedical Engineering, Shanghai Jiao Tong University, Shanghai 200240, China; mj.liu@sjtu.edu.cn (M.L.); shenyi12@sjtu.edu.cn (Y.S.); zfshao@sjtu.edu.cn (Z.S.)

**Keywords:** Atomic Force Microscopy, AFM, cellular elasticity, single molecule

## Abstract

One of the most successful applications of atomic force microscopy (AFM) in biology involves monitoring the effect of force on single biological molecules, often referred to as force spectroscopy. Such studies generally entail the application of pulling forces of different magnitudes and velocities upon individual molecules to resolve individualistic unfolding/separation pathways and the quantification of the force-dependent rate constants. However, a less recognized variation of this method, the application of compressive force, actually pre-dates many of these “tensile” force spectroscopic studies. Further, beyond being limited to the study of single molecules, these compressive force spectroscopic investigations have spanned samples as large as living cells to smaller, multi-molecular complexes such as viruses down to single protein molecules. Correspondingly, these studies have enabled the detailed characterization of individual cell states, subtle differences between seemingly identical viral structures, as well as the quantification of rate constants of functionally important, structural transitions in single proteins. Here, we briefly review some of the recent achievements that have been obtained with compressive force spectroscopy using AFM and highlight exciting areas of its future development.

## 1. Introduction

Atomic Force Microscopy (AFM) has made significant contributions to our understanding of biological systems in, arguably, two broad directions: imaging and force spectroscopy. For the former, the inherently high signal-to-noise ratio of AFM has been exploited to resolve sub-nanometer features of the surface topography of biological samples, notably proteins and DNA, directly from unprocessed images [1,2,3]. This has led to fundamental discoveries of membrane proteins [4,5], including pore-forming toxins [6,7,8,9], as well as of membrane-remodeling proteins [10], DNA-protein interactions [11,12,13], and antibodies [14], among other biological molecules [15]. Recently, the ability to obtain such high-resolution images at a much higher frame rate (down to tens of milliseconds per frame) with so-called high speed AFM has been demonstrated [16,17], which has enabled direct resolution of functionally important conformational changes of single biological molecules over physiologically relevant time-scales [18,19,20,21,22].

For the latter, AFM has played an instrumental role within the last few decades in the recognition of the important role of physical forces in biology [23]. In particular, the effects of forces on the structure and structural changes within biological molecules are now recognized to be critical to understand a wide range of basic biological functions [24,25,26]. These AFM studies generally entail the application of a tensile (or pulling) force on a single molecule that is subtended between the AFM tip and a solid substrate, while monitoring the consequences of this force by measuring the cantilever deflection as a function of time [1,26]. By varying the force magnitude and application velocity, subtle features of the underlying rate constants associated with the structural changes can be obtained [27,28]. Moreover, such single-molecule unfolding/separation trajectories reveal individualistic characterizations of the biomolecular conformational changes under force, thus providing information also about the (much more elusive) pathways of such events, including the detection of otherwise unknown intermediates [29]. From the groundbreaking studies of titin [30,31,32] and DNA [33,34,35] to the plethora of studies of membrane proteins [5,26], recently now shown at a remarkable microsecond timescale [36], force spectroscopy indeed remains one of the most powerful applications of AFM in biology.

However, from almost the inception of AFM in biology and well before the first tensile force spectroscopic (TFS) studies, it was commonly observed that compressive forces imparted by the tip during imaging can profoundly affect the structure of biological samples (usually recognized as a key reason limiting image quality) [1,37,38]. This led shortly after to the first application of AFM to study the elasticity of cells, as well as other biomaterials [39]. Over the years, elasticity measurements of cells have indeed been one of the most common applications of AFM in biology [40,41]. Following this initial work on cells, similar compressive force studies emerged of the slightly smaller biological complexes of viruses, whose mechanical properties are absolutely critical to their functioning [42,43,44,45,46]. Now, just within the last few years, compressive force investigations of single proteins have also been performed, providing direct quantitative characterizations of structural transitions that might not otherwise be known [47,48,49,50,51]. Overall, while these compressive force studies are not usually discussed at the same time as the more commonly known TFS applications, they can be clearly seen as their technical equivalent, only differing in the direction of the applied force. Interestingly though, while TFS has predominantly focused on only single biomolecules, compressive force spectroscopic (CFS) investigations have spanned samples whose length scale extends nearly three orders of magnitude, from single cells down to single proteins.

Here, we discuss some of the recent achievements of this lesser recognized application of AFM in biology, providing examples of its application that stretch from the very large to the very small.

## 2. Theoretical Considerations

Owing to the fact that most TFS studies focus on single biomolecules, the underlying theory used to interpret the data in different studies is generally similar, essentially describing the effects of force on transition barriers separating different states of the system [27,28]. By contrast, owing to the wide range of samples and length scales over which the force is applied in CFS studies, there is a broader range of theories that have been employed for their interpretation. Hence, before describing the results that have been obtained, we briefly describe some of the more common theoretical descriptions in CFS studies.

### 2.1. Elastic Theory for Macroscopic Biological Structures: Cells

**Figure 1:**
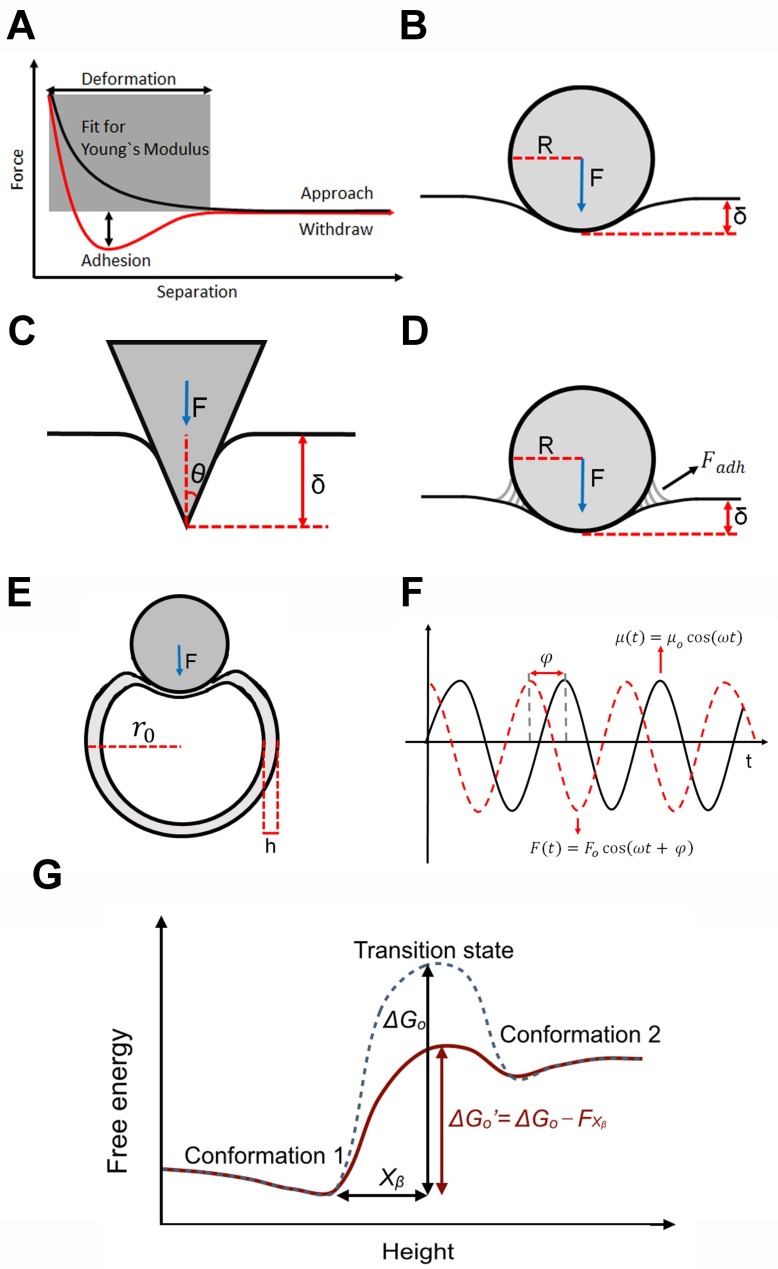
Overview of the theories generally used to interpret compressive force spectroscopic (CFS) data. (**A**) Schematic diagram of the experimental force versus distance curves showing the region of the data used for many mechanical measurements of biosamples; (**B**–**D**) Various models used to obtain a measure of the Young’s modulus of cells, including the Hertz model (**B**); Sneddon model (**C**); and the Derjaguin–Muller–Toporov (DMT) model (**D**); In each, the tip is colored grey and the indentation depth is δ; (**E**) Schematic diagram showing the analysis of the virus capsid as a thin spherical shell; (**F**) Depiction of the dynamic oscillatory model used to determine the visco-elasticity of cells; (**G**) Energy landscape model for the interpretation of single molecule CFS data.

When the force applied by the AFM tip is distributed over a length scale that is much larger than the typical size range of the sample constituents, the atomic/molecular details of the latter may be ignored and the sample treated as a continuous medium [39,52,53]. As such, the theory of elasticity developed for macroscopic solid bodies is most frequently employed in CFS studies of cells. This work can be divided into those for which the sample is assumed to deform simultaneously and proportionally to the applied force (that is, elastically) and those for which the sample re-arranges even under a steady force (that is, visco-elastically). To date, in most CFS studies of cells, the results have been interpreted as completely elastic. In these, the proportionality constant associated with the applied force and measured indentation depth is called the Young’s modulus, *E*, with the precise form of the relation depending on the geometry of the AFM tip (Figure 1). 

In particular, as the AFM tip is most often considered either as a sphere or a cone, the most frequent models employed to describe the data are either the Hertz model (spherical tip, Figure 1B) [1] or the Sneddon model (conical tip, Figure 1C):

(1) $$\mathrm{Hertz}\;\mathrm{model}:\;F=\frac{4E\delta^{3/2}\sqrt{R}}{3\left(1-v^{2}\right)}$$

(2) $$\mathrm{Sneddon}\;\mathrm{model}:\;F=\frac{2E\delta^{2}\tan\theta }{\pi \left(1-v^{2}\right)},$$

where *υ* is the Poisson ratio (assumed to be 0.5, that of an incompressible material), *F* is the applied force, δ is the indentation depth, *θ* is the half-opening angle of the conical tip, and *R* is the radius of the spherical tip. The radius of the tip (generally tens of nanometers for cells) and the half-opening angle (generally 15° to 40°) are usually measured using scanning electron microscopy. 

With some samples, there is also a significant adhesion force between the tip and the sample during the experiment, which thereby contributes to the overall applied force. In this case, the Derjaguin–Muller–Toporov (DMT) model, which assumes a spherical tip as with the Hertz model, is often employed (Figure 1D):

(3) $$\mathrm{DMT}\;\mathrm{model}:\;F=\frac{4E\delta^{3/2}\sqrt{R}}{3\left(1-v^{2}\right)}+F_{adh},$$

where $F_{adh}$ is the adhesion force measured from the force curve.

### 2.2. Elastic Theory for Macroscopic Biological Structures: Viruses

At sizes generally one to two orders of magnitude smaller than cells, viruses are still sufficiently larger than the typical sizes of AFM tips, and so, to a first approximation, can also be considered as continuous media and thus explicable using the theory of elasticity [53]. Further, the viruses are most often treated as elastic systems (at least for small deformations that do not permanently damage the virus, as described later).

Structurally, most viruses consist of a thin porous proteinaceous shell called the capsid, within which the genetic material (DNA or RNA) is contained [54,55]. Experimentally, most studies involve only empty viruses and it is found that at indentations greater than the thickness of the shell, there is an extended regime over which the force is linear with the indentation depth [56,57]. Within this regime thus, the virus capsid behaves like an ideal spring so that the AFM cantilever and capsid particle can be considered as two springs in series. Hence the measured effective spring constant, *k_m_*, is related to the virus spring constant, *k_v_*, and the spring constant of the cantilever, *k_c_*, by:

(4) $${k_{m}}^{-1}={k_{v}}^{-1}+{k_{c}}^{-1}\;\mathrm{or}$$

(5) $$k_{v}=\frac{k_{c}\;\times \;\;k_{m}}{k_{c}\;-\;k_{m}}$$

Treating the virus capsid as a thin spherical shell (Figure 1E), the virus spring constant is related to the Young’s modulus, *E*, by [45,53]:

(6) $$\mathrm{Thin}\;\mathrm{shell}\;\mathrm{model}:\;k_{v}=\frac{\alpha Eh^{2}}{r_{o}},$$

where *h* is the capsid thickness, *r_o_* is the capsid radius, and *α* is the geometry-dependent proportionality factor (shown to be ~1 in most cases [45,56,57]).

We note however that recent work has included a more sophisticated theoretical treatment including a thick shell and nonlinear spring model [58].

### 2.3. Viscoelastic Theory for Macroscopic Biological Structures

Although most CFS studies have assumed that the cell is elastic, detailed work by many different techniques has shown that the cell is actually visco-elastic [59,60,61,62,63,64,65]. Consistent with this, a recent CFS study, noting substantial disagreements between their measured Young’s moduli values and those obtained previously at different loading rates [66], confirmed a substantial dependence on the rate of force application, a hallmark sign of viscoelasticity [62,67,68]. Thus, the response of the cell is not precisely instantaneous with the application of force.

While not common, there have been CFS studies that explicitly probed this visco-elastic cell behavior [63,69,70,71,72]. In these, since the application of force is sinusoidal, the temporal difference between the applied force, *F*, and sample deformation, *μ*, manifests as a phase lag, *φ*, between the force and deformation (Figure 1F). With

(7) $$F\left(t\right)=F_{o}\cos(\omega t+\;\varphi )=Re\;\left[\hat{F}\left(\omega ,\;\varphi \right)]=Re\;[F_{o}\left(\omega \right)e^{i\varphi }\right]$$

and

(8) $$\mu \left(t\right)=\mu_{o}\cos(\omega t),$$

a complex elastic modulus, $\hat{G}\left(\omega \right)$, is defined as

(9) $$\hat{G}\left(\omega \right)=\frac{\hat{F}\left(\omega \right)}{\mu_{o}}=\frac{F_{o}\left(\omega \right)}{\mu_{o}}\left(\cos\varphi +i\;\sin\varphi \right)$$

which is then decomposed into real and imaginary components as

(10) $$\mathrm{Storage}\;\mathrm{modulus}:\;G\prime \left(\omega \right)=\frac{F_{o}\left(\omega \right)}{\mu_{o}}\cos\varphi \;(\mathrm{elastic}\;\mathrm{component})$$

(11) $$\mathrm{Loss}\;\mathrm{modulus}:\;G\prime \prime \left(\omega \right)=\frac{F_{o}\left(\omega \right)}{\mu_{o}}\sin\varphi \;(\mathrm{viscous}\;\mathrm{component})$$

### 2.4. Theoretical Treatment for Single Molecule Transitions: Rate Theory 

With some single molecule CFS studies, the applied force is found to induce a sudden, irreversible change in the structure of the protein that cannot be characterized by elastic theory. Instead, these changes resemble those often observed in TFS that are explained with reference to the energy landscape (Figure 1G). With this, there is an energy barrier separating structural states of the protein that can only be overcome by thermal fluctuations. 

We note that, unlike most ensemble-based experiments that entail equilibrium measurements, these single-molecule AFM experiments are often out-of-equilibrium, proceeding at rates faster than are necessary to enable the system to relax to equilibrium. Thus, at first brush, it may not be clear if AFM could be used to possibly determine the parameters of the energy landscape. However, Jarzynski showed that the free energy difference between two positions on this landscape can indeed be determined from non-equilibrium measurements of the work done in changing the system between these two states [73,74]. This result was confirmed experimentally [75] and further elaborated theoretically to a more generally applicable form for AFM measurements [27,28].

Crudely [76], the application of force, *F*, can be thought to reduce the height of the energy barrier, Δ*G_o_*, by an amount, *Fx_β_*, where *x_β_* is the reaction coordinate distance to the energy barrier peak from the minimum (Figure 1G). As a result, the protein overcomes this energy barrier by thermal fluctuations in a much shorter period of time than without the application of force. By varying the applied force, *F*, and monitoring the probability of the system to undergo this structural transition, the magnitude of the energy barrier, Δ*G_o_*, and the reaction coordinate distance, *x_β_*, can be determined.

In particular, if a constant force is applied for a specific length of time, *t*, this probability is given by

(12) $$P=1-\;e^{-k_{f}t},$$

where $k_{f}$, the force-dependent rate constant, and $k_{o}$, the force-free rate constant, are defined by

(13) $$k_{f}=\;k_{o}\times \;e^{\frac{Fx_{\beta }}{k_{B}T}}$$

(14) $$k_{o}=\;A\times \;e^{\frac{-\Delta G_{o}}{k_{B}T}},$$

*A* is the attempt frequency, *k_B_* is Boltzmann’s constant, and *T* is the temperature.

## 3. CFS of Cells: Elucidation of the Mechano-Phenotype

With the recent recognition of the significant role that force plays in biology has come the understanding that mechanical stimuli, whether the static stiffness of the extracellular matrix, the shear flow of blood or interstitial fluid, or the active physical forces imparted between cells, can all elicit a response from cells in much the same way as the more traditional biochemical stimuli [23,77]. As a result, the mechanical properties of cells are critical determinants for how cells respond to these mechanical stimuli, as they determine the degree of structural changes within the cell to which the cell can respond. Accordingly, these mechanical properties are as much an integrative measure of the cellular state as, say, the collection of transcripts or proteins within the cell, which are typical properties of the phenotype of the cell. Thus “mechano-phenotyping” of cells, that is the characterization of these mechanical properties, has emerged as a powerful label-free indicator of cell type and state for both basic biological and clinical applications.

### 3.1. Elastic Moduli of Cells: A General Characteristic of Cell Function 

**Figure 2:**
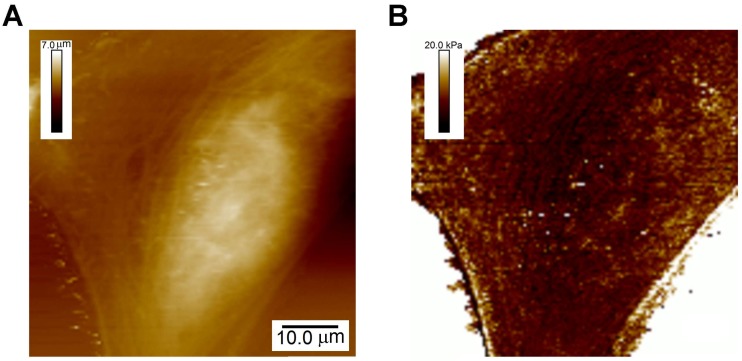
Characterization of the map of the Young’s modulus across live cells. (**A**) Topographic image of U2OS cells; (**B**) Map of the Young’s modulus across these cells acquired simultaneously.

As described above (Section 2.1), the Young’s modulus, *E*, is the proportionality constant between the extent of deformation and the applied force for purely elastic material. An example of a typical CFS measurement of the stiffness across a live cell is shown in Figure 2. It is by far the most commonly mechanical property that has been measured of cells and, though just a single parameter, has proven to be a remarkably useful indicator of cell function. 

#### 3.1.1. Elastic Moduli of Different Cell Types 

Table 1 shows the values of the Young’s modulus obtained by CFS for a wide range of cell types. Limiting analysis to the “normal” (that is, non-cancerous) cells, there is indeed a wide range of values, from those of neurons that are only a few kPa to cardiac cells that are a few hundred kPa.

Although we still lack a thorough quantitative understanding of the complete mechanisms underlying these measurements [65], it is generally believed that they are largely owing to the properties of the cytoskeletal constituents: filamentous actin (F-actin), microtubules, and intermediate filaments [65,78,79,80,81,82,83,84]. Of these, F-actin, together with its many binding proteins, form a cellular cortex immediately below the plasma membrane that is believed to contribute the most to the Young’s modulus of cells, at least for small deformations as in these CFS studies [65,85]. As such, the differences observed between different cell types might be expected to be primarily owing to the differences within this cortex, especially in the constituents of the actin-binding proteins [65].

As such, CFS studies of purified cytoskeletal components would be expected to usefully inform these measurements from cells. Indeed, detailed studies of the mechanical properties of actin networks have provided unexpected observations of their stiffening behavior that could be tested in the measurements with cells [86]. Further, such work with purified materials enables the elaboration of more detailed models of these highly complex filamentous systems [87,88].

Still, a recent study of the elasticity of both tissues and primary cells identified a striking correlation between the cellular elastic modulus and the nuclear lamin A concentration, as well as the levels of collagen in the extracellular matrixes of the tissues [89]. These results were interpreted as owing to the normal stresses experienced by the cells in situ, with tissues such as neurons being softer since they usually experience little stress compared to that of bone, which are harder and are more frequently under significant mechanical stress. Thus, while CFS measurements of the Young’s modulus might indeed be primarily owing to the cortex of these cells, they nonetheless appear to be associated with a more global, cell-wide state of the cell that reflects their physiological mechanical demands within the tissue [89].

#### 3.1.2. Elastic Moduli of Different Cell States 

Thus, as global measures of the cell, these types of CFS measurements might also be expected to reveal subtle differences between different states of the same cell type, whether occurring during normal biological processes such as development or as a result of the application of drugs.

Indeed, an interesting example of the former is the identification of striking differences in local mechanical properties during cell division [103]. While it was known that the local tension within the cell cortex is highest within the cleavage furrow, it was not known whether this was owing to a local increase in stiffness in the furrow or a decrease in stiffness at the poles. Taking advantage of the sub-cellular resolution in CFS, these authors showed that cortical stiffening occurs over the equatorial region actually about 160 s before any division furrow appears, and continues to rise as the furrow begins to form. However, this occurred without any consistent change to the stiffness within the polar regions. The authors suggested early signaling events, possibly produced from the spindle mid-plane, might initiate this increased equatorial local stiffening [104,105].

Another recent example of CFS to profile differences in cell states is a study that detailed incremental changes in cell stiffness during differentiation of myocytes [106,107,108]. Processes, as well as the differentiation of stem cells more broadly [97,109], have been observed to occur at different times within different cells within an isogenic population, and such studies provide a means to identify and characterize subtle differences in cell state that could be used to better understand and coordinate these transitions for biomedical applications [110].

#### 3.1.3. Use of Cell Elasticity Measurements in Cancer Diagnosis 

An extreme, and critically important, state that some cells can adopt is that of cancer, one of the major sources of mortality in the developed world. Early detection of cancer is often viewed as vital for successful treatment [111]. Often however, it is only later stages of cancer that are first detected, owing to the difficulty of visualizing small tumors (<1 mm^3^) or detecting biomarkers with sufficient specificity and sensitivity [112].

For reasons that are not clear though, one of the universal features of cancer cells studied to date is that they are substantially softer than their normal healthy counterparts [113,114]. This has been observed by CFS on many different cell types and in many different labs and instruments [66,94,102,115,116,117]. As such, there is great promise for this method in the early detection of cancer, especially as it is label free and exhibits cellular to sub-cellular resolution.

One of the best examples in this regard is a recent CFS study of human breast cancer biopsies [118]. In this work, the authors demonstrated clearly that the malignant cancer tissue exhibited a unique distribution of elasticity moduli significantly different from normal or even benign cancer tissue. As such, it is the distribution, and not simply a single measurement, that provides a nano-mechanical signature of the malignant tissue. These authors further showed significant intra-tumor variations in local stiffness that appeared to be associated with the extent of hypoxia experienced by the cells. Such information would not be evident from, for example, DNA sequencing or transcriptome analyses, but may prove important in determining how the cancer develops over time [118].

Additional studies have further demonstrated a strong correlation between the elastic moduli of cells and their metastatic potential [66,119,120], as well as the effects of drugs on the cells [121,122,123]. Interestingly though, it is not yet clear why cancer cells are softer, in terms of differences in the underlying molecular constituents. In general, actin is one of the most highly expressed proteins, even in cancers [124,125,126,127], and so a depletion of F-actin is unlikely to be responsible. This would also be consistent with the fact that many cancer cells are highly mobile, which requires a fully functional actin cortex [128,129,130,131,132]. Further, cancer is generally regarded as a genetic disease, arising from the accumulation of a sufficient number of “driver” mutations that confer a growth advantage to the cancer cell in which they occur [133]. What is the relationship between this growth advantage and a softer cell? Is there a “driver” mutation that leads to the softer cell? Or is a softer cell a symptom of the many other “passenger” mutations that invariably accumulate during cancer progression? A detailed understanding of the molecular reasons for this observation would provide information that could be exploited for novel therapeutic applications. 

### 3.2. Measurements of Viscoelastic Properties of Cells 

Notwithstanding these significant results, a wide range of studies have nonetheless shown that cells are inherently viscoelastic, with elastic moduli that depend on the rate of force application [63,65,66]. As such, a thorough knowledge of the mechano-phenotype of the cell, whether for diagnostic applications or to provide more detailed information to illume the mechanisms underlying cell elasticity, ultimately requires as complete a measurement of the CFS over as wide a range of frequencies as possible. 

Early efforts in this regard were limited to somewhat longer timescales, with frequencies between 0.01 to 100 Hz, in studies of live cells [63,70,134,135]. For example, a study of human lung epithelial cells over three frequency decades (0.1–100 Hz) with different loading forces showed that the storage modulus *G*′ increases weakly with frequency following a power law with exponent ~0.2, while the loss modulus *G*″ is ~2/3 lower and increases similar to *G*′ up to ~10 Hz, but shows a steeper rise at higher frequencies [63]. Interestingly, this behavior is consistent with that expected for soft glassy materials close to a glass transition, suggesting a highly precarious state of the cell cortex in general [63]. Further, the weak frequency dependence of *G*′ as well as the dominance of *G*′ over *G*″ at lower frequencies may provide an explanation for the utility of the measurements of Young’s moduli obtained assuming that the cell is a strictly elastic material, even though, strictly speaking, mammalian cells are visco-elastic. 

**Figure 3:**
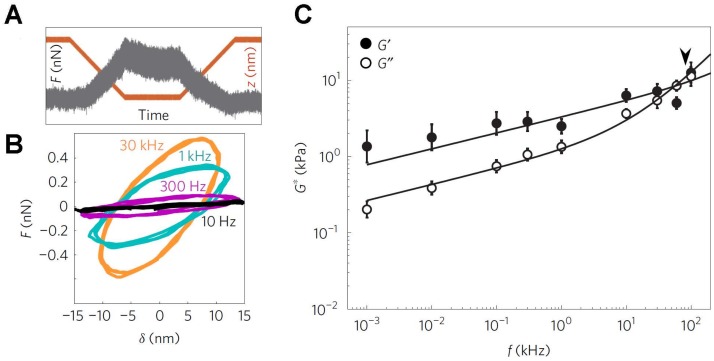
Measurement of the visco-elasticity of living cells over a broad range of application frequencies. (**A**) Typical force–time trace for the case with a 1 kHz oscillation obtained on a 3T3 fibroblast cell. Upon attaining an indentation of 250 nm, there is a small reduction in force, from a maximum of 0.5 nN, together with the superimposed oscillation; (**B**) The force–indentation cycles determined from the contact region of force curves exhibit significant differences at different oscillation frequencies, notably in the slope and degree of hysteresis in the cycle; (**C**) Frequency dependence of the storage modulus, *G*′, and loss modulus, *G*″, from the 3T3 cells. The transition frequency, separating the two regimes, is depicted by the arrowhead. This image is adapted from Rigato et al. published by Nature (2017) [137].

More recently, Scheuring and colleagues adapted their high-speed AFM that was developed for TFS [136] to enable high-frequency viscoelastic measurements from living cells, up to a maximum frequency of ~100 kHz (Figure 3) [137]. Interestingly, these authors identified two characteristic frequency regimes (Figure 3). At low frequencies, the response followed a weak power law as described above, but at high frequencies, the loss moduli *G*″ exhibited a steeper power law dependence (while the G′ remained similar to that of lower frequencies). The authors made the provocative suggestion that such a steeper rise in loss moduli is owing to the mechanical behavior of single F-actin filaments [137]. As it is possible to image the local F-actin distribution within cells using AFM, as well as identify regions devoid of extensive filaments [138], it should be possible to directly test this intriguing proposal in the future. We note that these authors also identified differences in the scaling dependencies between benign and malignant cancer cells at high frequencies. Thus, just as with elastic CFS studies, there appears to be great potential for visco-elastic CFS studies in the diagnosis and therapy of cancer. 

## 4. CFS Measurements of Sub-Cellular Structures 

One the main advantages of CFS over other techniques that can measure the mechanical properties of cells is the incredible range of sample sizes that can be investigated. Ultimately, this is limited by the size of the AFM tip, which can be as small as 2 nm for those commercially available. As such, there would appear to be many sub-cellular structures, spanning sizes from microns to hundreds of nanometers, such as organelles, endocytic vesicles, and ribosomes, that could be studied with CFS. To date though, no other biological complex within this size range has been studied as thoroughly with CFS as viruses. 

### Measurements of Elastic Properties of Viruses 

Viruses are remarkable self-assembled multi-molecular complexes whose structures span lengths from 20 nm to ~300 nm [54,55]. Overall, they all have a common architectural design in where a thin porous, proteinaceous shell encapsulates the genetic content. Further, the structure of these capsids has been found to be extremely economical: they are essentially only as large as needed to contain their nucleic acid at literally close-packing densities [139,140]. However, this extremely high packing density comes with significant energetic costs, both entropic and enthalpic, and so there is an extremely high internal pressure (tens of atmospheres) exerted by the tightly packed genetic material on the capsid structure [141,142]. As the capsid must withstand this internal pressure and maintain its structural integrity no matter the external conditions, it clearly must be exceedingly mechanically stiff. However, under the appropriate conditions, the capsid must also release the genetic content to the host cell where it will replicate. In addition, during the self-assembly process, the capsid proteins must be sufficiently flexible to enable the proteins to find their optimal interacting surfaces. That is, the capsid must also be soft, at least transiently. Most of the CFS studies to date have been designed to examine the underlying mechanisms associated with these intriguing, but apparently contradictory, mechanical requirements.

For example, work from many CFS studies has shown that most virus capsids are indeed quite stiff, exhibiting Young’s moduli roughly 10,000 to 100,000 times stronger than those mentioned above for cells (generally ~1 GPa) [143,144,145,146,147,148,149,150]. With this stiffness, an internal pressure of 60 atm would be expected to produce only a 3% increase in capsid radius (assuming a spherical shape) [53,141], and thus would only result in a very small degree of stretching within each capsid protein (assuming homogeneity in the stress distribution).

Further, it has been found that many viruses solve the soft/hard dichotomy by existing in two mechanically distinguishable states. For example, the bacteriophage HK97 capsid proteins self-assemble first into an immature structure (Prohead I) that exhibits a Young’s modulus of 0.3 GPa, which then transitions into a more rigid particle (Prohead II) with a Young’s modulus of 1 GPa, following the proteolytic removal of a capsid domain [151]. A similar increase in stiffness is observed during the packaging process of the λ bacteriophage as a result of the binding of gpD proteins, resulting in a 67% in stiffness [152]. 

Interestingly however, the human immunodeficiency virus type 1 (HIV-1) exhibits clear differences in mechanical strength during the maturation process but in the opposite direction, with Young’s moduli of 0.93 and 0.44 GPa for the immature and mature virion, respectively [153]. Such a lowering of stiffness was then found to correlate well with host cell entry [153]. This striking correlation suggests that there may be mechanical stresses generated within the virion during cell entry that precipitate the breakdown of the capsid so that, unless the capsid is sufficiently pliable, entry is prohibited. Still, it is not yet clear, in terms of a benefit for the virus, why the immature virion must be so much stiffer than the mature complex, particularly, as mentioned above, a greater stiffness would seem prohibitive to the self-assembly process. 

An additional mechanical observation in many these studies is that there is apparently a force at which the capsid structure is found to locally break down, akin to what is observed in macroscopic systems [144]. Changes in this breakdown force during virus maturation and assembly/disassembly often mirror those for the Young’s modulus [144,154], although providing additional information of the precise spatial location associated with the breakdown. In fact, the ability to resolve such a local structural change, involving only a few proteins, suggests that further CFS investigations at a single-protein level within these structures should be possible and highly informative to our understanding of the molecular-level details underlying the structural stability of these fascinating complexes. 

## 5. CFS of Single Proteins 

Finally, CFS has recently indeed been extended down to the study of individual protein and protein complexes [47,48,49,50]. As mentioned in the introduction, the effects of compressive force on imaging had been recognized since the earliest applications of AFM in biology [1,37,38,155]. Moreover, during the years, there have been anecdotal observations of an effect of a specific imaging force on the structure of single proteins [156,157]. However, for reasons that are not clear, more thorough studies of the effects of different forces on the detailed changes in protein structure have not appeared until the last few years. 

For one, local stiffness maps with spatial resolution down to 1–2 nm were obtained of the well-studied membrane protein, bacteriorhodopsin [50,51]. The ability to simultaneously measure the topography of the protein at this high resolution, together with its known atomic model, then enabled the direct correlation of the stiffness maps with the underlying secondary structures. The authors found that the α-helical transmembrane regions were associated with the greatest stiffness, while the extra-membranous loops connecting the α-helices were the softest, which might be expected. However, differences between the different helices and between the different loops, as well as the actual value of the stiffness of both (~80 pN/nm [50] corresponding to ~40 GPa assuming a tip radius of 2 nm) provide, perhaps, otherwise unattainable information about the mechanical properties of these proteins that aids our understanding of its functioning [50].

However, attributing observations such as these to a local stiffness may not always be valid, as this presumes a continuous transition between different structures under force. Instead, some proteins and protein complexes have been observed to undergo an abrupt change in structure under compressive force, with significant implications to their function [47,48,49].

In particular, a CFS study of the bacterial pore-forming toxin, perfringolysin O (PFO), characterized the mechanical details, and thereby the energetics, of an important structural transition of this protein, namely its pre-pore to pore transition [48]. This protein binds to membranes as monomers, then self-assembles into ring-shaped pre-pore complexes (containing ~36 subunits on average) that do not perforate the bilayer, which then insert into the bilayer to form the pore. Previous work had shown that this pre-pore to pore transition is associated with a remarkable 4 nm height collapse [7], but the energetic details of this collapse were poorly understood.

**Figure 4:**
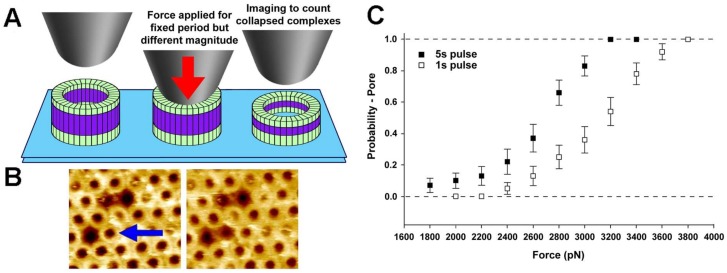
Single molecule CFS characterization of the pre-pore to pore transition in the pore-forming toxin, PFO. (**A**) Schematic diagram of the experiment, whereby a constant force in applied to pre-pore trapped complexes for a specific length of time (1 s or 5 s), followed by imaging at much lower forces to determine if the force was sufficient to catalyze the vertical collapse of the complex to the pore height; (**B**) Typical atomic force microscopy (AFM) images before (left) and after (right) the application of ~110 pN/monomer (~4000 pN) to a single pre-pore complex (blue arrow) Image size is 200 × 220 nm^2^; (**C**) Force and time dependence of the pre-pore to pore height collapse, from which details of the energy landscape associated with this transition can be directly obtained. This image is adapted from Czajkowsky et al. published by eLife (2015) [48].

This work showed that applying a compressive force to the top of pre-pore trapped complexes could catalyze the vertical collapse to the pore height (Figure 4). In particular, by applying a constant force to the complex for a fixed duration (1 s or 5 s), the force-dependence of this transition could be precisely detailed (Figure 4). Following the theory described above (Section 2.4), it was concluded that the structural change from pre-pore to pores is associated with an energy barrier of ~16 kcal/mol, which agreed well with all atom molecular dynamics simulations [48]. Thus, a barrier of this magnitude must be overcome during this transition, which was suggested to be mediated by the membrane insertion of the pore-lining region [48]. This combination of CFS and atomic-scale simulations is a powerful approach to detail conformational changes in biological molecules, as already well demonstrated in TFS [29,158].

## 6. Future Perspectives

As described here, CFS can indeed provide useful, functionally relevant information for an incredible wide range of biological samples, large and small. However, to date, these different types of studies were more frequently discussed separately, rather than within common framework as presented here.

We suggest that the usefulness of bringing together these disparate studies is that the physical processes studied in one area might be expected to be relevant also in the other areas, at least in some form. For example, the CFS studies with cells show the value of studying at different force application frequencies, particularly at high frequencies. However, to our knowledge, there have not been any extensive studies so far of the frequency dependence of the elasticity measurements of viruses. While breakdown of the viruses may limit the duration of such experiments, it would be interesting to determine whether or not there is a visco-elastic component to the mechanical properties, especially with the filled or partially filled capsids. Would the global properties of the virus dominate a low frequency regime while the local properties of the proteins and nucleic acid content dominate a higher frequency regime? Could the physical state of the closely packed genetic material and its interaction with the inner capsid surface be inferred? Likewise, the frequency-dependence of the single protein measurements could also provide useful information about the structural transitions, as these would enable direct testing of specific predictions from models based on the energy landscape [159,160]. 

Going in the other direction, from the small to the large, it would be interesting to determine whether fixed pulse experiments as described for PFO (Figure 4) could be performed with the viruses to characterize the breakdown event. Perhaps there is a similar sigmoidal relationship with the applied force that could then be associated with a specific energy barrier associated with the breaking of local secondary or tertiary structures within specific proteins. With cells, the softness of the cytoplasm might prohibit these types of experiments, though as mentioned above, the highest frequency regime has been suggested to be owing to the local mechanical behavior of single filaments, which could be tested. 

Hence, there indeed appear to be many potentially significant avenues of CFS in biology that appear to be technically feasible but have yet to be explored, beyond the many samples not yet investigated. We thus expect that the coming years will continue to show CFS as insightful and, perhaps more practically useful in clinical applications, than its presently more popular younger brother.

## Figures and Tables

**Table 1:** Young’ modulus of mammalian cells using AFM.

Cell Source	*E* (kPa)	Indentation (nm)	Loading Rate	Model	Reference
Rat Liver endothelial cells	2	n.r.	n.r.	Hertz	[90]
3T3 cells	3–12	<100	0.05 Hz	Sneddon	[91]
Neuronal growth cones–C domain	3–7	185	0.1 Hz	Hertz	[92]
Neuronal growth cones–T domain	7–23	101			
Neuronal growth cones–P domain	10–40	76			
Human SaOS2 osteoblast cell line	5.4–7.6	n.r.	0.2 Hz	Sneddon	[93]
Human cervix cell line End1/E6E7	5.5	<150	n.r.	Sneddon	[94]
Erythrocytes	19–33	200	n.r.	Hertz	[95]
Normal human urothelial cells	27.57	270~970	0.5 Hz	Sneddon	[96]
Human mesenchymal stem cells(hMSCs)	33	n.r.	10 Hz	Hertz	[97]
hMSCs–chondrocytes	39				
hMSCs–osteoblasts	52				
Human skin cell line	40	300~750	0.75 µm/s	Hertz	[83]
Skeletal muscle cells	21–28	80	0.25 µm/s	Sneddon	[69]
Cardiac cells	90–110				
Ovarian cancer cell line (high invasive)	0.494	1000	n.r.	Hertz	[98]
Ovarian cancer cell line (low invasive)	0.884				
Cancerous bladder epithelium cells	2.46	270~970	0.5 Hz	Sneddon	[96]
Hela cells	2.48	<150	n.r.	Sneddon	[94]
Human melanoma cell lines	0.421	500~1000	1 µm/s	Hertz	[99]
Esophageal cell lines	2.6	1216	2 µm/s	Hertz	[100]
Human colon cancer cell lines	0.479	1400	n.r.	Sneddon	[101]
Benign prostate tumors	3.03	500	8 µm/s	Sneddon	[102]
Aggressive prostate tumors	1.72				

n.r. = not reported.

## Abbreviations

AFM	Atomic force microscopy
TFS	Tensile force spectroscopy
CFS	Compressive force spectroscopy
DMT	Derjaguin–Muller–Toporov
